# The Effect of Foot Reflexology on Pain and Comfort During Labor: A Randomized Controlled Trial

**DOI:** 10.3390/healthcare14182981

**Published:** 2026-09-12

**Authors:** Nurgül Kılıçaslan, Esra Yurtsev

**Affiliations:** 1 Health Care Services Department, Istanbul Kanuni Sultan Suleyman Training and Research Hospital, Istanbul 34303, Turkey; nurgul.kilicaslan@saglik.gov.tr; 2 Department of Midwifery, Faculty of Health Sciences, Istanbul Topkapi University, Istanbul 34087, Turkey

**Keywords:** foot massage, reflexology, labor pain, labor comfort, intrapartum care, non-pharmacological methods

## Abstract

**Highlights:**

**What are the main findings?**

- Foot reflexology was associated with lower labor pain scores during the first stage of labor and higher comfort scores during the latent and active phases.
- Repeated application of foot reflexology across the latent, active, and transition phases was associated with phase-specific differences in pain and more frequent calm and controlled behavioral responses during labor.

**What are the implications of the main findings?**

- Foot reflexology may represent a feasible supportive non-pharmacological approach for pain management and comfort during the first stage of labor.
- Further studies should evaluate different application times and frequencies and confirm these findings in larger and more diverse populations.

**Abstract:**

**Background/Objectives**: Effective labor pain management and labor comfort are essential for a positive birth experience. Evidence regarding the effects of foot reflexology on labor pain and labor comfort remains limited. Therefore, this randomized controlled trial investigated the effects of foot reflexology on labor pain as the primary outcome and labor comfort as the secondary outcome. **Methods**: This prospective randomized controlled trial was conducted between February 2024 and February 2025 in the obstetrics and gynecology unit of a tertiary training and research hospital and was reported in accordance with the CONSORT 2025 guidelines. Seventy pregnant women were randomly assigned to the reflexology (*n* = 35) and control (*n* = 35) groups. Data were collected using structured forms, the Visual Analog Scale (VAS), and the Childbirth Comfort Questionnaire (CCQ) and analyzed using appropriate statistical procedures, with statistical significance set at *p* < 0.05. **Results**: Post-intervention pain scores were significantly lower in the reflexology group during the active (*p* = 0.009) and transition phases (*p* < 0.001). Complementary linear mixed-effects analyses showed significant group × time interactions for pain across the latent, active, and transition phases (all *p* < 0.001), indicating greater pre-to-post reductions in the reflexology group. The standardized post-intervention between-group differences were small, moderate, and large in the latent, active, and transition phases, respectively. Labor comfort was significantly higher in the reflexology group during the latent (*p* = 0.008) and active phases (*p* = 0.044), and calm and controlled behavior was observed more frequently in the reflexology group (*p* = 0.03). **Conclusions**: Foot reflexology was associated with reduced labor pain and improved comfort during the first stage of labor. Repeated application across the latent, active, and transition phases showed differential reductions in pain across labor phases, suggesting that foot reflexology may be a feasible supportive non-pharmacological intervention during labor.

## 1. Introduction

Labor pain is a distinctive form of acute pain that varies considerably in intensity among women [1]. Effective pain management during labor is recommended by the American College of Obstetricians and Gynecologists (ACOG) and is considered a fundamental component of quality obstetric care [2]. Accordingly, effective management of labor pain is recognized as an important goal of intrapartum care [3].

In addition to effective pain management, the WHO emphasizes reducing discomfort and promoting comfort as important components of a positive childbirth experience [4]. Kolcaba conceptualizes comfort as a holistic and multidimensional construct encompassing physical, psychospiritual, sociocultural, and environmental dimensions [5]. Within this framework, comfort during labor is not determined solely by the relief of physical pain but also reflects women’s psychological and social experiences and may be influenced by sociocultural and environmental factors [6,7]. Thus, although labor pain and comfort may be interrelated, they represent distinct constructs. Effective management of labor pain may promote relaxation and support women’s ability to cope during labor. Accordingly, pain management should extend beyond pain relief to support coping and comfort, which may contribute to a more positive, empowering, and holistic childbirth experience [6,8].

Although these two concepts are interrelated during the birth process, they can also be considered independently of one another when necessary. A woman’s perception of comfort during labor may not correspond directly to pain intensity; even when pain is severe, relatively high levels of comfort may still be experienced [9]. Reduced labor pain may be associated with greater comfort and a more positive perception of the childbirth experience, while comfort-enhancing interventions may support women’s ability to cope with labor pain [10,11]. Accordingly, midwifery care plays an important role in promoting comfort during childbirth, including through the use of non-pharmacological approaches to support labor pain management [8].

The WHO guidelines on intrapartum care emphasize that non-pharmacological methods, such as massage, can improve the childbirth experience by reducing labor pain and alleviating discomfort during childbirth [4]. Non-pharmacological methods are widely recognized as potentially effective, safe, and low-risk approaches for the management of labor pain [4,12]. The effectiveness of massage, one of these methods, in reducing labor pain has been demonstrated in various studies. Various massage-based approaches are used for pain management during labor [11,13]. Among these massage-based approaches, foot massage follows the general principles of massage, whereas foot reflexology differs in its specific theoretical basis and application principles. Reflexology, a form of manual therapy-based massage, is a method with roots dating back to ancient times that is still used today [13]. According to traditional reflexology theory, specific areas of the hands and feet are believed to correspond to different parts or organs of the body. These reflex zones, however, reflect the theoretical framework of traditional reflexology rather than established anatomical or physiological connections [14]. Unlike general foot massage, foot reflexology involves targeted and systematic pressure applied to specific areas of the foot, referred to as reflex zones within reflexology practice [14]. Although the physiological mechanisms underlying reflexology have not been conclusively established, theoretical explanations include stimulation of peripheral sensory pathways through pressure applied to the hands and feet, with subsequent responses related to stress reduction, relaxation, and pain modulation [15,16,17]. Gate control theory has also been discussed as a potential explanation for pain modulation associated with touch- and massage-based interventions [18]. Different approaches to reflexology are used in practice. The Ingham method involves only hand and finger techniques without auxiliary tools, whereas the Rwo Shur method uses auxiliary tools resembling wooden sticks [14]. Today, foot reflexology is increasingly used as a complementary approach in various clinical settings [19]. It has been used during pregnancy and the postpartum period to address symptoms such as nausea, vomiting, leg swelling, fatigue, and breastfeeding difficulties, as well as during childbirth to reduce anxiety and pain [17]. Studies have also reported potential benefits of foot reflexology for pain management in various conditions, including musculoskeletal, postoperative, pregnancy-related, childbirth-related, chronic, and cancer-related pain [14,17]. In particular, foot reflexology has been reported as a potentially useful complementary approach for reducing labor pain [14,17]. However, the overall evidence remains limited, and relatively few studies have specifically examined its effect on labor comfort. Therefore, this study aimed to evaluate the effect of foot reflexology on labor pain and labor comfort among nulliparous women. Labor pain was considered the primary outcome, while labor comfort was considered the secondary outcome. Accordingly, the following hypotheses were tested:

**H1.**  *Foot reflexology reduces labor pain intensity in nulliparous women compared with routine care.*

**H2.**  *Foot reflexology increases labor comfort in nulliparous women compared with routine care.*

## 2. Materials and Methods

### 2.1. Purpose and Type of Study

This experimental, prospective, randomized controlled clinical trial was conducted to evaluate the effectiveness of foot reflexology during the first stage of labor in reducing perceived labor pain and improving labor comfort.

### 2.2. Study Setting and Participants

The study was conducted in a tertiary training and research hospital. In accordance with institutional policy, companions were not permitted in the labor ward; therefore, participants did not have access to continuous one-to-one labor support such as doula care or similar support persons. All participants received standard midwifery care as part of routine clinical practice. This care included admission to the labor ward, monitoring during the first stage of labor in collaboration with the obstetric physician, assistance during birth, initial neonatal assessment, and postpartum follow-up during the first two hours after birth.

Non-pharmacological methods for labor pain relief were not routinely provided in the institution. Within the scope of this study, foot reflexology was administered only to participants in the intervention group. Pharmacological agents for pain management could be used based on clinical indication and at the discretion of the attending physician; however, no pharmacological analgesic agents were administered for labor pain relief to participants in either the intervention or control groups during the study period.

The onset of labor and the latent phase were determined based on the presence of regular painful uterine contractions accompanied by progressive cervical changes, including cervical effacement and dilatation. Consistent with the WHO definition, women presenting with these clinical findings and cervical dilatation of <5 cm at enrollment were considered to be in the latent phase of the first stage of labor [4].

The study included pregnant women with full-term pregnancies (between 37 and 42 weeks of gestation), with a single, live fetus in vertex presentation, who were in the latent phase of labor with regular uterine contractions and progressive cervical changes, had cervical dilatation of less than 5 cm at enrollment, were planning a vaginal birth, had no pregnancy complications, had no language problems, were able to communicate verbally, and agreed to participate in the study. Pregnant women using narcotic analgesics or sedatives, those with systemic diseases such as heart disease, hypertension, diabetes, or kidney disease, and those with pregnancy complications such as placenta previa, preeclampsia, oligohydramnios, polyhydramnios, or fetal malpresentation were excluded from the study.

### 2.3. The Population and Sample of the Study

The study population consisted of nulliparous pregnant women planning vaginal birth who presented to the labor room of the hospital where the study was conducted between February 2024 and February 2025. During this period, a total of 671 nulliparous pregnant women who met the inclusion criteria were screened.

Sample size was calculated using G*Power 3.1.9.7 based on the expected between-group difference in VAS pain scores. An expected effect size of Cohen’s d = 0.68, derived from the mean and standard deviation values of VAS scores reported in the reference study, was used for the calculation. With a two-sided α level of 0.05, 80% statistical power, and a planned 1:1 group ratio, the minimum required sample size was calculated as 70 participants (35 per group). Considering potential attrition, additional participants were recruited. A total of 83 eligible women were randomized, of whom 40 were allocated to the reflexology group and 43 to the control group. Following post-allocation losses, the final analysis included 70 participants, with 35 women in each group.

### 2.4. Data Collection Tools

#### 2.4.1. Demographic Information Form

This form was developed based on a literature review and was used to assess the sociodemographic characteristics, medical and obstetric histories, and knowledge and attitudes regarding childbirth among pregnant women. The form consists of questions that assess sociodemographic characteristics, obstetric and medical history, and knowledge and attitudes regarding childbirth, as well as perceptions of labor pain, the level of knowledge regarding non-pharmacological methods used to alleviate labor pain, and awareness of foot reflexology massage [8,20].

#### 2.4.2. Labor Monitoring Form

In monitoring the labor process, a Labor Monitoring Form was used, prepared based on the literature. The form included gestational age, the date and time of onset of labor contractions, medications and interventions applied during the first stage of labor, pre- and post-reflexology assessments (cervical dilation and effacement, fetal head station, membrane status, frequency and intensity of uterine contractions, fetal heart rate, maternal vital signs, and maternal behaviors during labor), the start and end times of the second stage of labor, and the total duration of labor [20].

#### 2.4.3. Visual Analog Scale (VAS)

Labor pain intensity was the primary outcome of the study and was assessed using the Visual Analog Scale (VAS). The Visual Analog Scale (VAS) is a 0–10 scale used to assess pain intensity, with endpoints representing ‘no pain’ and ‘worst imaginable pain.’ Participants indicate their current pain level on the scale, which is typically presented horizontally [21,22]. For participants who were unable to read the scale instructions, the use of the VAS and its endpoints were explained verbally by the researcher, and participants indicated their own perceived pain intensity.

#### 2.4.4. Childbirth Comfort Questionnaire (CCQ)

Labor comfort was the secondary outcome of the study and was assessed using the Childbirth Comfort Questionnaire (CCQ). The CCQ was developed by Schuiling (2003) [23] and adapted into Turkish by Potur et al. (2015) [24]. It is a 9-item, 5-point Likert-type scale with total scores ranging from 9 to 45, with higher scores indicating greater comfort. The reported Cronbach’s alpha coefficient is 0.75 [24], while in the present study, Cronbach’s alpha values ranged from 0.818 to 0.838 across the labor phases. For participants who were unable to read the questionnaire, the items and response options were read aloud by the researcher without interpretation or guidance, and the participants’ responses were recorded accordingly.

### 2.5. Statistical Analysis

Descriptive statistics were presented as numbers, percentages, means, standard deviations, minimums, and maximums, as appropriate. Normality was assessed using the Shapiro–Wilk test and visual inspection. Between-group and within-group comparisons were performed using independent-samples *t*-tests and paired-samples *t*-tests, respectively. Categorical variables were compared using Pearson’s chi-square or Fisher’s exact test, as appropriate.

Changes in pain and comfort scores were evaluated using change-score analyses, with between-group differences compared using independent samples *t*-tests. Because baseline VAS scores differed between groups during the latent and active phases, ANCOVA was additionally used to compare post-intervention VAS scores after adjustment for the corresponding baseline values; the same approach was applied to the transition phase for consistency. ANCOVA assumptions were assessed. Linearity and residual diagnostics were generally acceptable; however, the homogeneity of regression slopes assumption was not met in the latent and active phases. Therefore, complementary linear mixed-effects models with group × time interactions were performed to account for repeated measurements within participants. The mixed-effects models were estimated using restricted maximum likelihood with an unstructured covariance structure (Supplementary Table S1).

Standardized between-group differences in VAS scores were expressed as Hedges’ g. Effect estimates were reported with 95% confidence intervals (CIs) to facilitate interpretation of their precision [25]. In accordance with recent recommendations for pain research, absolute Hedges’ g values of approximately 0.10, 0.30, and 0.70 were interpreted as tentative thresholds for small, medium, and large effects, respectively [26]. ANCOVA effect sizes were reported as partial eta squared (partial η^2^). Internal consistency was assessed using Cronbach’s alpha. The final analysis included 70 participants (35 per group). All analyses were performed using IBM SPSS Statistics for Windows, version 27.0 (IBM Corp., Armonk, NY, USA), with statistical significance set at *p* < 0.05.

### 2.6. Randomization and Allocation

Participants were randomly assigned to either the reflexology or control group using a computer-generated randomization sequence created through Randomizer.org. The randomization sequence was generated by the researcher before participant enrollment and allocation. Eligible participants who provided informed consent were assigned sequential participant numbers according to their order of enrollment, and group allocation was determined according to the corresponding assignment in the pre-generated randomization sequence. A total of 83 participants were randomized, of whom 40 were allocated to the reflexology group and 43 to the control group.

### 2.7. Procedure

**Figure 1:**
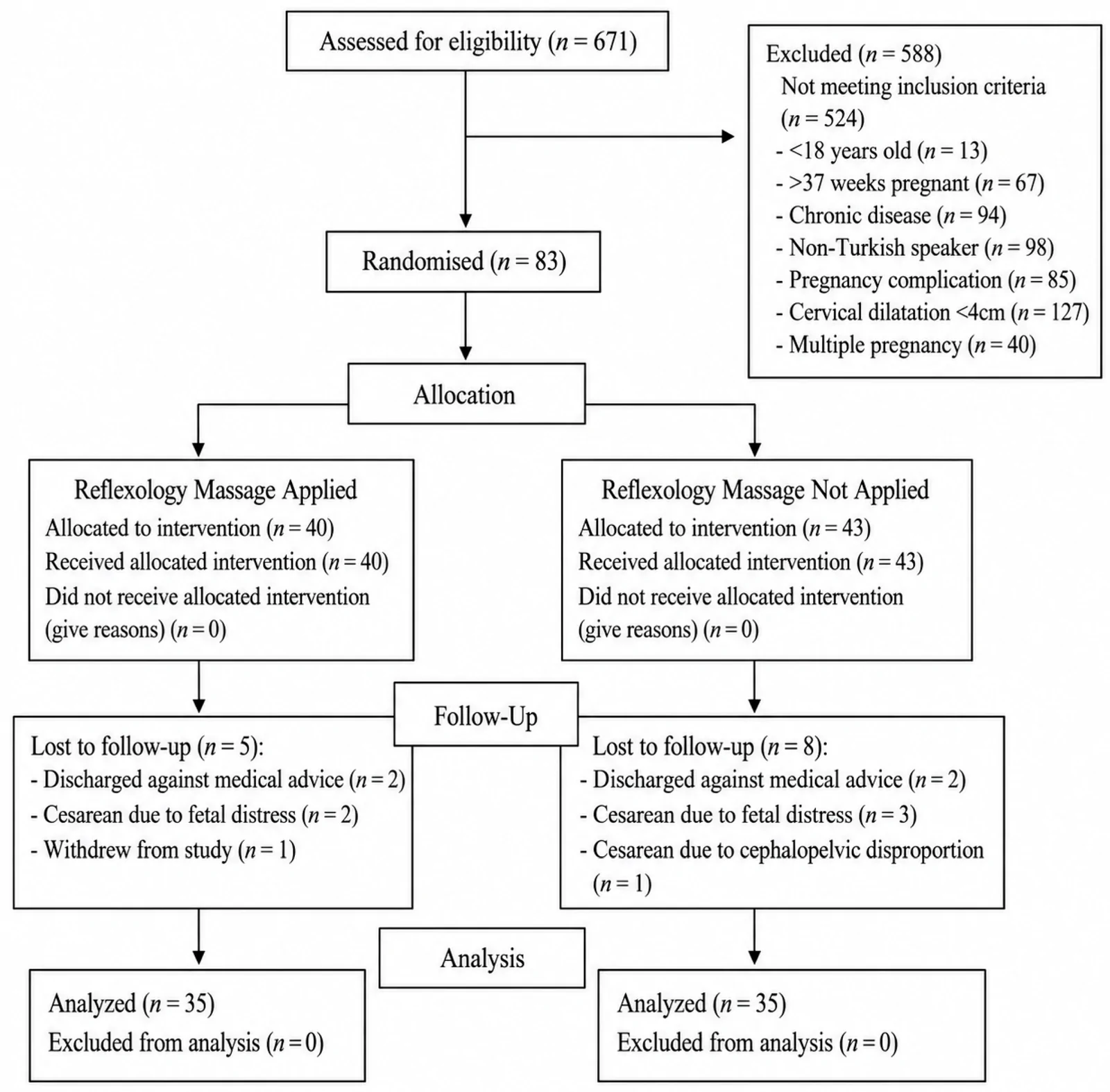
CONSORT Flow Diagram of Participant Recruitment and Allocation.

In the reflexology group, the intervention was applied after randomization by a researcher certified in clinical reflexology. The study adhered to CONSORT guidelines [27], and the flow diagram is presented in Figure 1.

**Figure 2:**
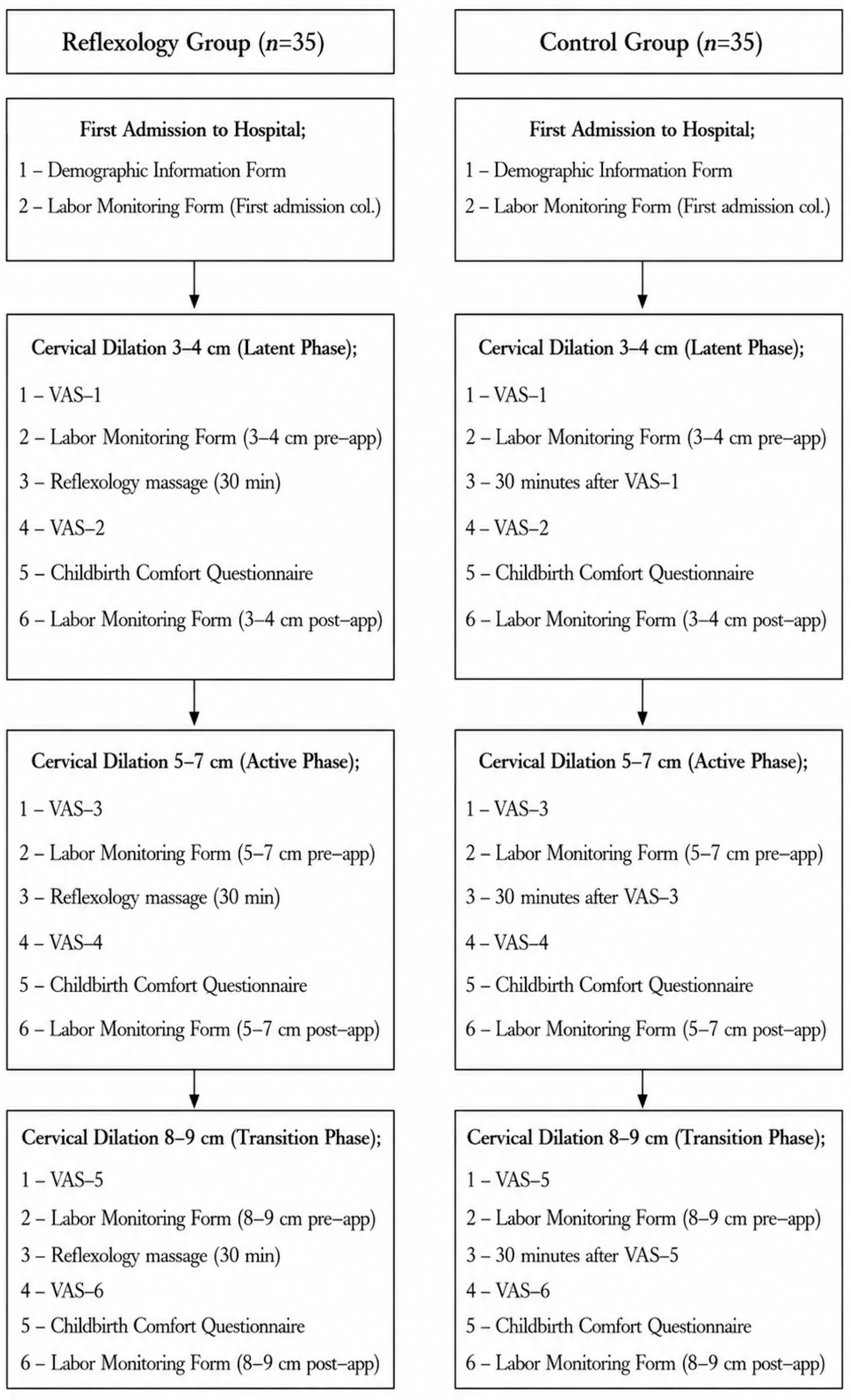
Study Flow Diagram.

The data were collected by the researcher through face-to-face interviews, clinical observations, and follow-up records throughout the first stage of labor. First, the Demographic Information Form and the Labor Monitoring Form were completed, detailing the findings upon arrival at the hospital. Both groups received standard care throughout the labor process in accordance with the hospital’s standard protocols. The study flow diagram is presented in Figure 2.

In the study, the intensity of contractions was assessed using cardiotocography (CTG) monitoring and clinically interpreted by both the attending physician and the midwife. Fetal heart rate parameters were assessed using CTG in terms of baseline heart rate, variability, accelerations, and decelerations.

**Figure 3:**
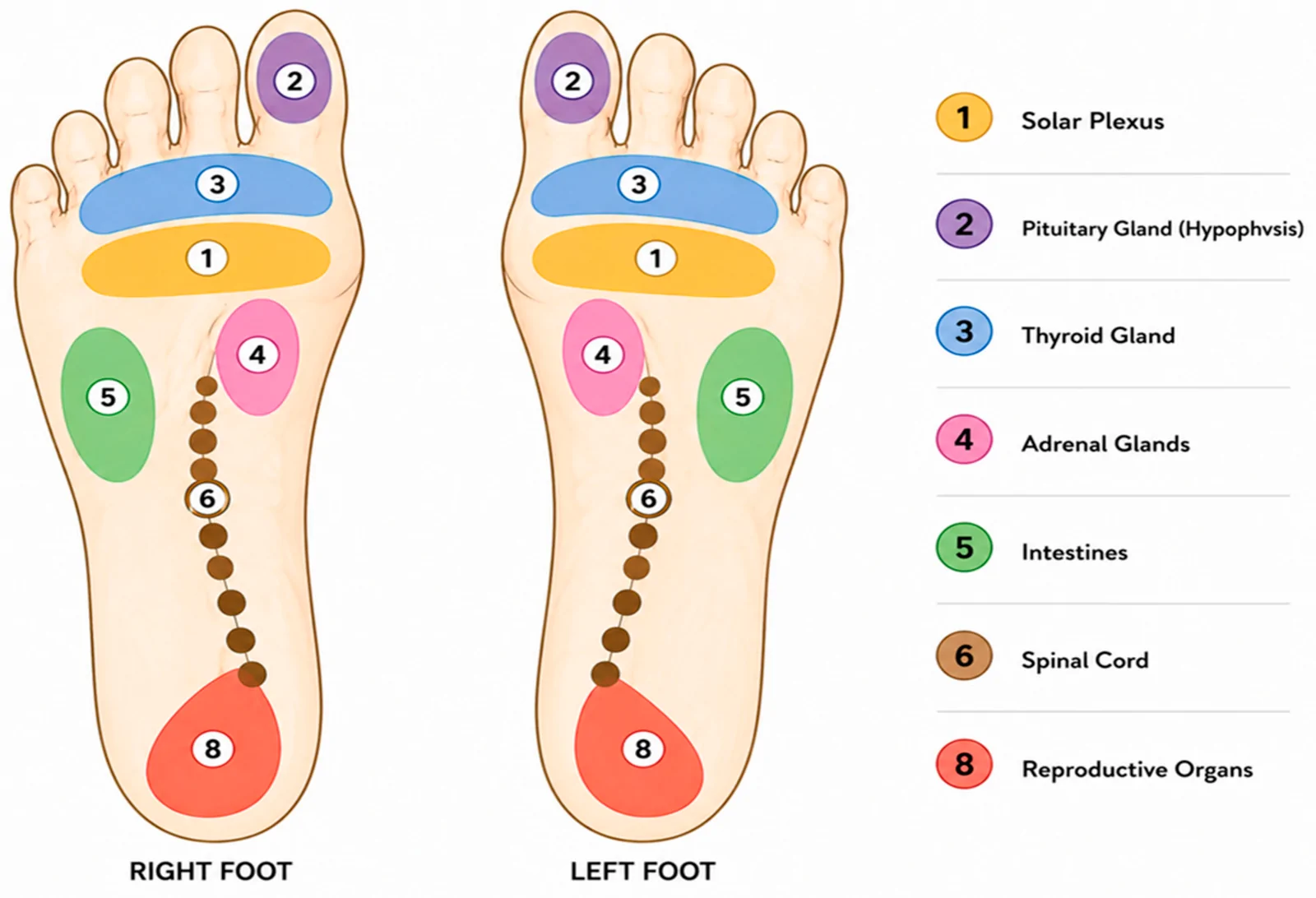
Reflexology Points Applied During the Intervention.

In the reflexology group, the intervention was administered at the beginning of each phase of the first stage of labor: at cervical dilations of 3–4 cm (latent phase), 5–7 cm (active phase), and 8–9 cm (transition phase). Before each session, pain intensity was assessed using the VAS (VAS-1, VAS-3, and VAS-5, respectively). Foot reflexology was then applied for a total of 30 min, with 15 min devoted to each foot. All reflexology sessions were performed by the same researcher, who had received reflexology training before the study, to ensure consistency in the application of the intervention. A standardized protocol was followed across all participants and labor phases. Using manual thumb-pressure techniques, pressure was applied systematically to the predefined reflex points associated with the solar plexus, pituitary gland, thyroid gland, adrenal glands, intestines, spinal cord, and reproductive organs, following the sequence illustrated in Figure 3 [16,28,29]. Pressure intensity was not instrumentally quantified; however, consistency of application was maintained by having the same trained researcher use the same manual thumb-pressure technique and standardized sequence throughout all sessions. Particular attention was given to the reflex points included in the protocol for pain management during labor. The same sequence and duration were maintained for both feet and repeated during each of the three labor phases. Each session ended upon completion of the standardized 30-min protocol. Immediately after each session, post-intervention pain intensity (VAS-2, VAS-4, and VAS-6, respectively), CCQ scores, and follow-up data were recorded (see Figure 2). Due to the nature of the intervention, participants, the intervention provider, and outcome assessors were not blinded to group allocation; however, statistical analyses were performed by an independent statistician who was blinded to group allocation.

In the control group, no intervention was applied; VAS measurements were conducted at the same dilation points, with second measurements obtained 30 min later (VAS-2, VAS-4, VAS-6). CCQ and follow-up forms were completed accordingly (see Figure 2).

## 3. Results

Of the 83 participants randomized, 13 did not complete the study. In the reflexology group, 2 participants declined the intervention, 2 underwent cesarean delivery due to fetal distress, and 1 withdrew from the study. In the control group, 2 participants declined participation, 3 underwent cesarean delivery due to fetal distress, 1 underwent cesarean delivery due to cephalopelvic disproportion, and 2 withdrew from the study. The final analysis included 70 nulliparous women, with 35 participants in each group. Post-randomization attrition was 12.5% (5/40) in the reflexology group and 18.6% (8/43) in the control group, with no statistically significant difference between groups (Fisher’s exact test, *p* = 0.551). Participant flow is presented in the CONSORT diagram (Figure 1).

### 3.1. Demographic and Obstetric Findings

The mean age was 24.49 ± 4.74 in the reflexology group and 23.54 ± 3.70 in the control group, with no significant difference (*p* = 0.36). The groups were similar in terms of sociodemographic and obstetric characteristics (Table 1 and Table 2).

Participants’ perceptions of labor pain and their knowledge levels regarding pain relief methods are presented in Table 3. No statistically significant difference was found between the reflexology and control groups in terms of the distribution of pain perception (*p* = 0.89). Similarly, no statistically significant difference was found between the groups in terms of knowledge levels regarding pain relief methods (*p* = 0.30).

### 3.2. Labor Pain and Comfort Outcomes

#### 3.2.1. Labor Pain Across the Phases of the First Stage of Labor

Labor pain outcomes were evaluated using complementary analytical approaches addressing different aspects of the intervention effect. Phase-specific change-score analyses were used to quantify between-group differences in pre-to-post changes, while ANCOVA provided baseline-adjusted comparisons of post-intervention VAS scores and linear mixed-effects models were used to further evaluate group × time effects across repeated measurements.

Women’s general views on labor pain were similar across groups, with approximately half of the participants describing labor pain as very severe or unbearable (Table 3). Before foot reflexology during the latent phase, VAS pain scores were significantly higher in the reflexology group than in the control group (*p* = 0.004; Table 4). After foot reflexology, however, no significant difference was observed between the groups (*p* = 0.33; Table 4). During the active phase, pre-intervention VAS pain scores were also significantly higher in the reflexology group than in the control group (*p* = 0.005). However, 30 min after the intervention, VAS pain scores were significantly lower in the reflexology group than in the control group (*p* = 0.009; Table 4). During the transition phase, pre-intervention VAS pain scores were similar between the groups (*p* = 0.24). In contrast, 30 min after the intervention, VAS pain scores were significantly lower in the reflexology group than in the control group (*p* < 0.001; Table 4).

The standardized between-group differences in post-intervention VAS scores corresponded to Hedges’ g values of −0.231, −0.635, and −1.467 in the latent, active, and transition phases, respectively (Table 4). According to the tentative thresholds proposed for pain research, these differences represented small, moderate, and large effects, respectively.

To account for baseline differences in pain scores, post-intervention VAS scores were additionally analyzed using ANCOVA, with the corresponding pre-intervention VAS score included as a covariate. ANCOVA showed significant adjusted between-group differences during the latent (F = 13.275, *p* < 0.001, partial η^2^ = 0.165), active (F = 30.312, *p* < 0.001, partial η^2^ = 0.311), and transition phases (F = 48.683, *p* < 0.001, partial η^2^ = 0.421), with adjusted means and 95% confidence intervals presented in Table 5. However, assessment of ANCOVA assumptions indicated that the homogeneity of regression slopes assumption was not met during the latent and active phases. Accordingly, the ANCOVA findings for these phases were interpreted with caution, with greater interpretative emphasis placed on the corresponding group × time effects derived from the linear mixed-effects models. Complementary linear mixed-effects analyses showed significant group × time interactions during the latent (F(1, 68) = 23.118, *p* < 0.001), active (F(1, 68) = 41.111, *p* < 0.001), and transition phases (F(1, 68) = 49.281, *p* < 0.001) (Supplementary Table S1).

#### 3.2.2. Labor Comfort Across the Phases of the First Stage of Labor

CCQ scores were significantly higher in the reflexology group than in the control group in the latent phase (30.11 for the reflexology group and 27.57 for the control group; *p* = 0.008) and in the active phase (29.63 for the reflexology group and 27.54 for the control group; *p* = 0.044). Although scores remained higher in the transition phase (28.06 for the reflexology group and 26.34 for the control group), the difference was not statistically significant (*p* = 0.07; Table 6).

The rate of calm and controlled behavior in the reflexology group was found to be significantly higher than that in the control group (*p* = 0.03; Table 7).

## 4. Discussion

This study examined the effect of foot reflexology on labor pain and comfort during childbirth. The sociodemographic and obstetric characteristics of the reflexology and control groups were comparable (Table 1 and Table 2). When the findings on labor pain and childbirth comfort were considered together, differences between the reflexology and control groups were observed among participants who completed follow-up. However, the potential influence of post-randomization attrition on the magnitude of the observed intervention effects was also taken into account when interpreting the findings.

### 4.1. Effects of Reflexology on Labor Pain

When the effect of foot reflexology on labor pain was evaluated across the latent, active, and transition phases of the first stage of labor, reductions in pain intensity were observed across all three phases. Baseline pain scores differed between groups during the latent and active phases; therefore, ANCOVA was initially used to account for these differences. However, because the homogeneity of regression slopes assumption was not met in these phases, complementary linear mixed-effects analyses were performed. These analyses demonstrated significant group × time interactions across all three phases, indicating that changes in pain over time differed between the reflexology and control groups. Importantly, the magnitude of the standardized post-intervention between-group differences increased across labor phases, from small in the latent phase to moderate in the active phase and large in the transition phase. Thus, the findings should be interpreted not only in terms of statistical significance but also according to the magnitude and precision of the observed effects. This phase-specific pattern is consistent with previous studies suggesting that the effects of foot reflexology on labor pain may vary across different phases of labor [30,31,32].

At the beginning of the latent phase, pain intensity was significantly higher in the reflexology group than in the control group (*p* = 0.004). After foot reflexology, this initial between-group difference was no longer statistically significant (*p* = 0.33; Table 4). Although ANCOVA indicated a significant adjusted between-group difference (F = 13.275, *p* < 0.001, partial η^2^ = 0.165; Table 5), the homogeneity of regression slopes assumption was not met. Accordingly, greater interpretative emphasis was placed on the complementary linear mixed-effects analysis, which demonstrated a significant group × time interaction (F(1, 68) = 23.118, *p* < 0.001), indicating that the pre-to-post pattern of change differed significantly between groups (Supplementary Table S1). Nevertheless, the standardized post-intervention between-group difference based on Hedges’ g was small. Taken together, these findings suggest that reflexology was associated with a differential change in pain during the latent phase, but the small magnitude of the post-intervention difference limits conclusions regarding its clinical importance at this stage.

Previous studies have reported that foot reflexology is most commonly administered as a single session during the active phase of labor [17,33], whereas in the present study the active-phase assessment followed a second reflexology session after the initial application during the latent phase. Previous studies have also reported reductions in labor pain following foot reflexology [15,17,31,32,34]. Consistent with this evidence, pain intensity in the reflexology group, although significantly higher at the beginning of the active phase (*p* = 0.005), was significantly lower than that in the control group after the intervention (*p* = 0.009; Table 4). ANCOVA also indicated a significant adjusted difference (F = 30.312, *p* < 0.001, partial η^2^ = 0.311; Table 5); however, because the homogeneity of regression slopes assumption was not met, greater emphasis was placed on the complementary mixed-effects analysis. The significant group × time interaction (F(1, 68) = 41.111, *p* < 0.001) demonstrated that the pattern of pain change differed between groups (Supplementary Table S1). Together with the moderate Hedges’ g for the post-intervention difference, these findings suggest that the observed active-phase effect may have potential clinical relevance. However, the magnitude of an effect alone does not establish clinical significance, and this interpretation should be considered in the context of the study design and its limitations.

During the transition phase, baseline pain intensity was similar between groups (*p* = 0.24), whereas post-intervention pain intensity was significantly lower in the reflexology group (*p* < 0.001; Table 4). In this phase, the homogeneity of regression slopes assumption was satisfied, and ANCOVA demonstrated a significant adjusted between-group difference (F = 48.683, *p* < 0.001, partial η^2^ = 0.421; Table 5). The complementary linear mixed-effects analysis similarly demonstrated a significant group × time interaction (F(1, 68) = 49.281, *p* < 0.001), indicating a differential change in pain between groups (Supplementary Table S1). Moreover, the transition phase showed the largest standardized post-intervention between-group difference, with a large Hedges’ g. Considering that the transition phase is characterized by particularly intense pain and increasingly frequent and strong contractions [35], the combination of a significant differential change and a large standardized difference suggests that the observed effect during this phase may have particular clinical relevance. Nevertheless, clinical relevance should be interpreted cautiously given the sample size and other methodological limitations of the study.

The pattern observed across labor phases is broadly consistent with previous evidence indicating that foot reflexology may reduce labor pain [17,31,32,34]. However, the present findings extend this evidence by examining repeated applications across three consecutive phases rather than evaluating a single application at one stage of labor. The progressive increase in the magnitude of the post-intervention between-group differences, from small in the latent phase to moderate in the active phase and large in the transition phase, may suggest that the observed effect varies according to the phase of labor or repeated exposure to the intervention. Because the present design does not allow the effects of labor phase and cumulative exposure to reflexology to be separated, however, this pattern should not be interpreted as evidence of a cumulative or dose–response effect. Further studies directly comparing different application frequencies and timing protocols are needed to clarify this issue.

The observed reductions in pain may be considered in relation to several physiological mechanisms proposed for manually applied non-pharmacological interventions [36,37]. General foot massage has been suggested to promote relaxation and modulate pain transmission in accordance with gate control theory [36]. Foot reflexology is also a touch-based intervention; however, unlike general foot massage, it involves systematic stimulation of reflex points on the foot that are proposed to correspond to specific organs and systems [16]. According to proposed nerve-impulse mechanisms, stimulation of these areas may influence autonomic activity and reduce physiological responses associated with stress and tension, thereby potentially modifying pain perception [31]. Similarly, although acupressure involves pressure applied to defined points, reflexology involves systematic stimulation of reflex zones across the foot [38,39]. These proposed mechanisms may provide possible explanations for the observed findings; however, the present study did not directly measure neurophysiological or autonomic mechanisms, and therefore no specific mechanism can be established from these data.

Another proposed distinction between foot reflexology and other manual techniques relates to the relationship between reflex points and fascial planes. The plantar fascia forms part of a broader fascial network, and stimulation of the foot has been proposed to influence tension and structural relationships beyond the local area [40]. Such mechanisms have been suggested as a possible explanation for broader physiological responses to reflexology. However, these explanations remain theoretical in the context of the present study and should not be interpreted as demonstrated mechanisms underlying the observed reduction in labor pain.

An important methodological feature of this study was the sequential application of foot reflexology during the latent, active, and transition phases rather than its administration at a single time point. This approach enabled the intervention to be evaluated across the progression of the first stage of labor, during which pain intensity normally increases. The significant group x time interactions across all three phases, together with the increasing magnitude of the standardized post-intervention differences, suggest that the association between reflexology and pain reduction was not confined to a single phase of labor. These findings contribute to the existing literature by providing phase-specific estimates of the observed effects of repeated reflexology application. Future trials directly comparing single and repeated applications are needed to determine whether intervention timing, frequency, or cumulative exposure influences the magnitude of the observed effect.

### 4.2. Effects of Reflexology on Labor Comfort

When evaluating the effect of foot reflexology on labor comfort, comfort scores were significantly higher in the reflexology group than in the control group during the latent (*p* = 0.008) and active (*p* = 0.044) phases. In the transition phase, although comfort scores were higher in the reflexology group, this difference did not reach statistical significance (*p* = 0.07; Table 6). Pain and comfort are two closely related concepts during the labor process. In this study, the evaluation of higher levels of comfort observed during the latent and active phases, in conjunction with the significant reduction in pain achieved following foot reflexology during the same phases, suggests that effective management of labor pain may help women perceive the labor process as more tolerable and manageable. However, given the multidimensional nature of comfort, it should also be considered that, unlike the control group receiving standard care, the touch, researcher attention, and supportive interaction accompanying the intervention in the reflexology group may have contributed to women’s perceived comfort.

Although comfort scores were higher in the reflexology group during the transition phase, the difference between the groups was not found to be statistically significant. This stage of labor is characterized by the most frequent and intense contractions, resulting in a marked increase in physical and emotional stress [36,41]. For this reason, it is thought that even though the pain-relieving effect of foot reflexology persists, it may not have had the same level of impact on women’s overall perception of comfort during this period. The literature also reports that inadequate management of labor pain can negatively affect women’s comfort and birth experience, whereas the support and care provided during labor are important factors in determining comfort [8,41]. In this regard, it is thought that the increased physiological and emotional burden during the transition phase may have limited the higher comfort scores observed in the reflexology group from reaching statistical significance.

The literature shows that massage techniques reduce pain and anxiety during childbirth, making the birthing experience more tolerable for women [42,43]. Although the number of studies directly assessing comfort during childbirth is limited, improvements in pain, relaxation, and satisfaction with the birth are considered important indicators of comfort [44,45,46]. When viewed from this perspective, the increase in comfort observed in our study, particularly during the latent and active phases, suggests that foot reflexology may serve as a complementary, non-pharmacological midwifery intervention capable of supporting women’s physical and psychological well-being during the birthing process.

When the findings are evaluated within the framework of Kolcaba’s Theory of Comfort, it becomes evident that comfort during childbirth is not limited to physical comfort alone; rather, it is viewed as a multidimensional construct encompassing physical, psychospiritual, sociocultural, and environmental dimensions [23]. This theoretical approach makes it possible to evaluate the behavioral responses exhibited by the reflexology group, which experienced a higher level of comfort, during childbirth. Thus, it helps to interpret the effect of foot reflexology on comfort during childbirth within a more holistic framework [11]. When evaluating the behavioral responses exhibited by women during childbirth, it was determined that “calm and controlled” behaviors were observed more frequently in the reflexology group, whereas completely uncontrolled or harmful behaviors were not observed at all (Table 7). This finding suggests that the effect of foot reflexology is not limited to the reduction in physical pain but may also support women’s emotional and cognitive self-regulation processes. In addition to the reduction in pain levels, the observation of calmer and more controlled behavioral patterns appears to be consistent with the “relief” dimension defined in Kolcaba’s Comfort Theory. The absence of completely uncontrolled or harmful behaviors in the reflexology group suggests that women were able to respond to the birthing process in a more balanced and harmonious manner, reflecting the “ease” dimension. Similarly, the fact that women were able to participate in the birthing process in a more controlled manner and regulate their behavior suggests that they were able to cope more effectively with labor, which also appears to align with the “transcendence” dimension.

The findings from our study are consistent with the results of previous studies. In a study evaluating the effects of massage administered during labor, it was reported that women who received touch and massage therapy felt more comfortable as labor progressed [36]. Similarly, Şanlı and Satılmış (2023) reported that negative behavioral reactions were less common among women who received classical foot massage during labor [47]. In their study based on Comfort Theory, Gupta et al. (2023) demonstrated that foot massage administered during the first stage of labor increased women’s positive behavioral responses [45]. In addition, it is known that enhancing women’s comfort during labor contributes to their developing more positive memories of the birth experience [11]. The literature also reports that increased comfort during labor has positive effects on pain intensity, anxiety levels, duration of labor, preference for vaginal delivery, and newborn health; and that women’s perception of comfort is one of the key factors determining satisfaction with labor [48,49,50]. When evaluated together, the findings related to pain, comfort, and behavioral responses suggest that foot reflexology may serve as a supportive non-pharmacological intervention during labor, with potential contributions to pain reduction, perceived comfort, and more positive behavioral responses.

## 5. Limitations

This study has several limitations. First, although ethical approval was obtained and the study protocol and outcomes were established before participant recruitment, the trial was registered retrospectively, which may raise concerns regarding reporting transparency and potential selective outcome reporting. Second, the single-center design and inclusion of only nulliparous women may limit the generalizability of the findings. Third, participant and outcome-assessor blinding was not feasible, and no sham reflexology or attention-control condition was used. Because pain and labor comfort were subjective outcomes, expectation and performance effects cannot be excluded, and the observed effects cannot be attributed exclusively to reflexology-specific mechanisms. Non-specific effects of touch, researcher attention, relaxation, supportive interaction, or participant expectations may also have contributed to the findings. Future trials should consider sham reflexology or attention-control conditions to distinguish specific from non-specific intervention effects. Finally, 13 randomized participants (reflexology, *n* = 5; control, *n* = 8) did not complete follow-up because of emergency cesarean delivery, refusal of the assigned intervention, or withdrawal. Consequently, complete-case analysis was used, and a full intention-to-treat analysis could not be performed because post-intervention outcome data were unavailable. This post-randomization attrition may have introduced attrition bias and potentially influenced the estimated magnitude of the intervention effect, as outcomes among participants who did not complete the study may have differed systematically from those of participants included in the complete-case analysis, potentially leading to either overestimation or underestimation of the observed effects.

## 6. Conclusions

The findings of this study indicate that foot reflexology was associated with reduced labor pain during the first stage of labor among nulliparous women. The magnitude of the post-intervention differences varied across labor phases, with a small effect observed in the latent phase, a moderate effect in the active phase, and a large effect in the transition phase. The moderate and large effects observed during the active and transition phases suggest that the pain reduction associated with foot reflexology may have potential clinical relevance, whereas the smaller effect observed during the latent phase warrants more cautious interpretation. In addition, foot reflexology was associated with improved comfort during the latent and active phases and with more frequent calm and controlled behavioral responses during labor. An important aspect of this study was the repeated application and evaluation of foot reflexology during the latent, active, and transition phases, allowing its effects to be examined across different phases of the first stage of labor. Overall, under the conditions of this trial, foot reflexology was associated with lower pain scores and higher comfort scores and may represent a feasible supportive non-pharmacological intervention for nulliparous women during the first stage of labor. These findings should nevertheless be interpreted in light of the absence of participant and outcome-assessor blinding and a sham or attention-control condition, which precludes excluding the contribution of non-specific intervention effects. Future studies should confirm these findings in larger and more diverse populations and compare different application times and frequencies to determine the most appropriate application protocols and their clinical relevance.

## Figures and Tables

**Table 1:** Sociodemographic Characteristics of Reflexology and Control Groups.

Characteristic	Reflexology (*n* = 35) *n* (%)	Control (*n* = 35) *n* (%)	Total (*n* = 70) *n* (%)	*p* Value
Mean Age	24.49 ± 4.74 (min: 18, max: 42)	23.54 ± 3.70 (min: 18, max: 34)		0.36 ^a^
Marital status				
Single	0 (0)	4 (11.4)	4 (5.7)	0.12 ^b^
Married	35 (100)	31 (88.6)	66 (94.3)	
Educational status				
No education	1 (2.9)	6 (17.1)	7 (10)	
Primary education	14 (40)	17 (48.6)	31 (44.3)	0.12 ^c^
High school	13 (37.1)	8 (22.9)	21 (30)	
University	7 (20)	4 (11.4)	11 (15.7)	
Partner’s educational status				
No education	1 (2.9)	1 (2.9)	2 (2.9)	
Primary education	10 (28.6)	20 (57.1)	30 (42.9)	0.10 ^b^
High school	15 (42.9)	10 (28.6)	25 (35.7)	
University	9 (25.7)	4 (11.4)	13 (18.6)	
Income				
Income less than expenses	7 (20)	8 (22.9)	15 (21.4)	
Income more than expenses	2 (5.7)	0 (0)	2 (2.9)	0.35 ^b^
Income equal to expenses	26 (74.3)	27 (77.1)	53 (75.7)	
Family structure				
Nuclear family	28 (80)	29 (82.9)	57 (81.4)	
Extended family	7 (20)	5 (14.3)	12 (17.1)	0.51 ^b^
Living alone	0 (0)	1 (2.9)	1 (1.4)	

^a^ Independent-samples *t*-test, ^b^ Fisher’s exact test; ^c^ Pearson’s chi-square test.

**Table 2:** Obstetric Characteristics of Reflexology and Control Groups.

Number of Pregnancies	Reflexology *(n* = 35)	Control (*n* = 35)	Total *(n* = 70)	*p* Value
	*n* (%)	*n* (%)	*n* (%)	
1.	30 (85.7)	28 (80)	58 (82.9)	0.79 ^a^
2.	4 (11.4)	6 (17.1)	10 (14.3)	
3.	1 (2.9)	1 (2.9)	2 (2.9)	
History of miscarriage				
Yes	5 (14.3)	7 (20.0)	12 (17.1)	0.54 ^b^
No	30 (85.7)	28 (80.0)	58 (82.9)	
History of abortion				
Yes	2 (5.7)	5 (14.3)	7 (10)	0.43 ^b^
No	33 (94.3)	30 (85.7)	63 (90.0)	
Planned pregnancy				
Yes	29 (82)	28 (80)	57 (81.4)	0.98 ^b^
No	6 (17.1)	7 (20)	13 (18.6)	
Experiencing any problem during pregnancy *				
Yes	1 (2.9)	3 (8.6)	4 (5.7)	0.61 ^a^
No	34 (97.1)	32 (91.4)	66 (94.3)	
Use of Medication During Pregnancy **				
Yes	4 (11.4)	2 (5.7)	6 (8.6)	0.67 ^b^
No	31 (88)	33 (94.3)	64 (91.4)	
Attending Routine Prenatal Care Appointments				
Yes	34 (97.1)	35 (100)	69 (98.6)	0.99 ^a^
No	1 (2.9)	0 (0)	1 (1.4)	

^a^ Fisher’s exact test; ^b^ Pearson’s chi-square test. * Mild pregnancy-related symptoms (nausea, fatigue, etc.) were not included in the assessment; only clinically diagnosed complications were recorded. ** The use of medications during pregnancy refers solely to medications prescribed for medical reasons; routinely used iron and vitamin supplements are not included in this category.

**Table 3:** Opinions of the Reflexology and Control Groups on Labor Pain and Knowledge of Reflexology.

General Opinion About Labor Pain	Reflexology (*n* = 35) *n* (%)	Control (*n* = 35) *n* (%)	Total (*n* = 70) *n* (%)	*p* Value
Very severe	17 (48.6)	16 (45.7)	33 (47.1)	0.89 ^a^
Unbearable	6 (17.1)	7 (20)	13 (18.6)	
Mild	1 (2.9)	1 (2.9)	2 (2.9)	
Disturbing	1 (2.9)	0 (0)	1 (1.4)	
Severe	10 (28.6)	11 (31.4)	21 (30)	
Hearing about methods to relieve labor pain				
Yes	13 (37.1)	8 (22.9)	21 (30)	0.30 ^b^
No	22 (62.9)	27 (77.1)	49 (70)	
Hearing about reflexology massage applied to feet				
Yes	4 (11.4)	2 (5.7)	6 (8.6)	0.68 ^b^
No	31 (88.6)	33 (94.3)	64 (91.4)	

^a^ Fisher’s exact test; ^b^ Pearson’s chi-square test.

**Table 4:** VAS Pain Scores Across the Phases of the First Stage of Labor in the Reflexology and Control Groups.

Labor Phase	Group	Mean	SD	t	*p* Value	Hedges’ g
VAS 1 Latent phase (pre-intervention/usual care)	Reflexology	6.51	2.490	2.963	**0.004**	**0.700**
	Control	4.94	1.909			
VAS 2 Latent phase (30 min post-intervention/usual care)	Reflexology	4.83	2.022	−0.990	0.33	**−0.231**
	Control	5.31	2.083			
VAS 3 Active phase (pre-intervention/usual care)	Reflexology	8.26	1.615	2.92	**0.005**	**0.695**
	Control	7.11	1.659			
VAS 4 Active phase (30 min post-intervention/usual care)	Reflexology	6.26	1.669	−2.699	**0.009**	**−0.635**
	Control	7.34	1.697			
VAS 5 Transition phase (pre-intervention/usual care)	Reflexology	9.49	0.702	1.196	0.24	**0.285**
	Control	9.26	0.886			
VAS 6 Transition phase (30 min post-intervention/usual care)	Reflexology	8.03	1.200	−6.228	**<0.001**	**−1.467**
	Control	9.51	0.742			

Pre-intervention measurements were obtained at the beginning of each labor phase. Post-intervention measurements were obtained 30 min later, following foot reflexology in the intervention group and routine care in the control group. Hedges’ g was calculated as Reflexology−Control; negative values indicate lower VAS scores in the reflexology group.

**Table 5:** Baseline-Adjusted Comparison of Post-Intervention VAS Pain Scores Between the Reflexology and Control Groups.

Labor Phase	Covariate	Adjusted Mean (Reflexology)	95% CI	Adjusted Mean (Control)	95% CI	F	*p*	Partial η^2^
Latent	VAS1	4.384	3.867–4.902	5.759	5.241–6.276	13.275	<0.001	0.165
Active	VAS3	5.908	5.462–6.355	7.692	7.245–8.138	30.312	<0.001	0.311
Transition	VAS5	8.196	7.919–8.473	9.575	9.298–9.852	48.683	<0.001	0.421

VAS, Visual Analog Scale; CI, confidence interval. Adjusted means were estimated using AN-COVA after controlling for the corresponding baseline pain scores. Effect sizes are presented as partial eta squared (partial η^2^).

**Table 6:** Childbirth Comfort Questionnaire (CCQ) Scores Across the Phases of the First Stage of Labor in the Reflexology and Control Groups.

Labor Phase	Group	Mean	SD	t	*p* Value
CCQ (Latent)	Reflexology	30.11	4.25	2.733	**0.008 ^a^**
	Control	27.57	3.50		
CCQ (Active)	Reflexology	29.63	3.57	2.054	**0.044 ^a^**
	Control	27.54	4.83		
CCQ (Transition)	Reflexology	28.06	3.46	1.83	0.07 ^a^
	Control	26.34	4.32		

^a^ Independent-samples *t*-test.

**Table 7:** Comparison of Behavioral Patterns During Labor Between the Reflexology and Control Groups.

Behavior Status	Group	Yes *n* (%)	No *n* (%)	Total	*p* Value
Calm and controlled	Reflexology	20 (57.1)	15 (42.9)	35	0.03 ^a^
	Control	12 (34.3)	23 (65.7)	35	
Quiet crying/agitated	Reflexology	17 (48.6)	18 (51.4)	35	0.63 ^a^
	Control	14 (40.0)	21 (60.0)	35	
Loud crying/difficulty maintaining control	Reflexology	13 (37.1)	22 (62.9)	35	0.80 ^a^
	Control	11 (31.4)	24 (68.6)	35	
Uncontrolled/potentially harmful behaviors	Reflexology	0 (0.0)	35 (100.0)	35	0.24 ^b^
	Control	3 (8.6)	32 (91.4)	35	

^a^ Fisher’s exact test; ^b^ Pearson’s chi-square test.

## Data Availability

The data presented in this study are available on request from the corresponding author. The data are not publicly available due to privacy and ethical restrictions.

## Supplementary Materials

The following supporting information can be downloaded at: https://www.mdpi.com/article/10.3390/healthcare14182981/s1. Table S1. Linear Mixed-Effects Model Analysis of Changes in VAS Pain Scores Across the Phases of the First Stage of Labor.
