# Supplementary material for: The Effect of Foot Reflexology on Pain and Comfort During Labor: A Randomized Controlled Trial
Source: Healthcare (Basel). 2026 Sep 12;14(18):2981. doi: 10.3390/healthcare14182981 (PMC13606597; doi:10.3390/healthcare14182981)
Supplement: Supplementary file 1 [file healthcare-14-02981-s001.zip › healthcare-4471527-supplementary.pdf]

Supplementary Table S1. Linear Mixed-Effects Model Analysis of Changes in VAS Pain Scores Across the Phases of the First Stage of Labor

Labor phase	Group	Pre-intervention EMM ± SE (95% CI)	Post-intervention EMM ± SE (95% CI)	Estimated change (Post−Pre)	Post-intervention between-group difference (95% CI)	Group × Time F	p
**Latent**	Reflexology	6.486 ± .379 (5.729–7.243)	4.857 ± .341 (4.176–5.538)	−1.629	− .429 (−1.392 to .535)	**23.118**	**< .001**
	Control	4.943 ± .379 (4.186–5.700)	5.286 ± .341 (4.604–5.967)	+ .343			
**Active**	Reflexology	8.171 ± .280 (7.613–8.730)	6.257 ± .284 (5.690–6.825)	−1.914	−1.086 (−1.888 to − .283)	**41.111**	**< .001**
	Control	7.114 ± .280 (6.556–7.673)	7.343 ± .284 (6.775–7.910)	+ .229			
**Transition**	Reflexology	9.514 ± .129 (9.257–9.771)	8.257 ± .150 (7.958–8.556)	−1.257	−1.257 (−1.680 to − .834)	**49.281**	**< .001**
	Control	9.257 ± .129 (9.000–9.514)	9.514 ± .150 (9.215–9.813)	+ .257			

[a]EMM, estimated marginal mean; SE, standard error; CI, confidence interval; VAS, Visual Analog Scale. Group 1 = reflexology; Group 2 = control. Estimated change was calculated as post-intervention minus pre-intervention EMM. Negative values indicate a reduction in pain. The between-group difference represents Reflexology − Control at the post-intervention assessment. Linear mixed-effects models included group, time, and the group × time interaction as fixed effects. A significant group × time interaction indicates that the change in VAS pain scores over time differed between groups.
